# The complete chloroplast genome of *Coelogyne barbata* (Orchidaceae), a rare and ornamental orchid

**DOI:** 10.1080/23802359.2020.1835569

**Published:** 2020-11-13

**Authors:** Yao Zhao, Qing-Qin Tan, Hui-Jun Guo, Lu Li

**Affiliations:** a National Plateau Wetlands Research Center, Southwest Forestry University, Kunming, China; b Department of Biodiversity Conservation, Southwest Forestry University, Kunming, China; c Department of Life Science, Southwest Forestry University, Kunming, China; d Southwest Forestry University, Kunming, China

**Keywords:** *Coelogyne barbata*, chloroplast genome, Orchidaceae, phylogenetic analysis

## Abstract

The genus of *Coelogyne* Lindl. comprised about 200 species while its generic relationship has been uncertain. The whole chloroplast genome of *C. barbata* was reported in order to provide new data on the molecular phylogeny of *Coelogyne*. The cp genome of *C. barbata* was 1,600,93 bp in total length, including a pair of inverted repeat regions (IR, 26,710 bp), one large single-copy region (LSC, 87,868 bp), and one small single-copy region (SSC, 188,05 bp). The complete chloroplast DNA encoded 132 genes, containing 86 protein-coding genes, 38 tRNA genes, and eight rRNA genes. Phylogenetic analysis showed that *C. barbata* was related to *Pholidota imbricata*.

The genus of *Coelogyne* Lindl. (tribe Arethuseae, subfam. Epidendroideae) comprised about 200 species in which generic relationship had been uncertain (Chase et al. 2015). *C. barbata* Lindl. ex Griff. was recognized by possessing sparse pseudobulbs with two leaves, hysteranthous inflorescence with white flowers, and margin long fimbriate on the lip. It grew on trees in forests or on cliffs and was distributed in SW China, Bhutan, NE India, and Nepal (Chen and Clayton 2009). Since the plastid genome would provide molecular evidence for plant systematic (Jansen et al. 2007; Nock et al. 2011; Pan et al. 2019), the complete chloroplast genome of *C. barbata* was reported.

Leaf samples of *C. barbata* were obtained from Fumin County, Yunnan Province, China (25°20′19″N, 102°27′26″E). The voucher specimen was deposited in the Herbarium of Southwest Forestry University (HSFU, Lilu-20180004). High quality chloroplast genomic DNA of *C. barbata* was extracted from the fresh leaf by using the modified CTAB procedure (Doyle and Doyle 1987) and sequenced on the Illumina Hiseq 2500 platform (Illumina, San Diego, CA) in Shanghai Personal Biotechnology Co., Ltd. (Shanghai, China). Genome sequences were assembled and screened using GetOrganelle pipe-line (Jin et al. 2018). Then, the new sequence was annotated with Geneious Prime version 2020.1.2 (Kearse et al. 2012). Finally, the chloroplast genome sequence was submitted to GenBank and obtained an access number MT627604.

The complete chloroplast genome of *C. barbata* was 160,093 bp in length and contained two inverted repeats (IR, 26,710 bp) regions, a large single-copy region (LSC, 87,868 bp), and a small single-copy region (SSC, 18,805 bp). This gene sequence encoded 132 genes, including 86 protein-coding genes, 38 tRNA genes, and eight rRNA genes. Among them, LSC, SSC, and IR regions accounted for 35.2%, 30.5%, and 43.3% of the GC-content. In addition, the overall GC-content was 37.3%. The gene order and organization of *C. barbata* were consistent with those of *Dendrobium* species in subfamily Epidendroideae (Wang et al. 2020).

To confirm the phylogenetic position of *C. barbata*, a maximum likelihood (ML) tree was conducted using RAxML v8.1.11 (Stamatakis 2014) as implemented on the Cyberinfrastructure for Phylogenetic Research (CIPRES) Science Gateway (http://www.phylo.org/, Miller et al. 2010), employing the GTRtG model with 1000 bootstrap iterations (-#/-N). Other parameters used the default settings. Phylogenetic analysis based on 79 protein-coding genes of 10 representative plastomes within the subfamily Epidendroideae as ingroup. According to the updated molecular phylogeny of Orchidaceae (Chase et al. 2015; Li et al. 2016), 10 species from two tribes (Malaxideae Lindl. and Arethuseae Lindl.) in subfamily Epidendroideae were selected as ingroup, while two species from subfamily Orchidoideae as outgroup. It turned out that *C. barbata* was closely related to *Pholidota imbricata* Hook. with 100% support (Figure 1).

**Figure 1.**
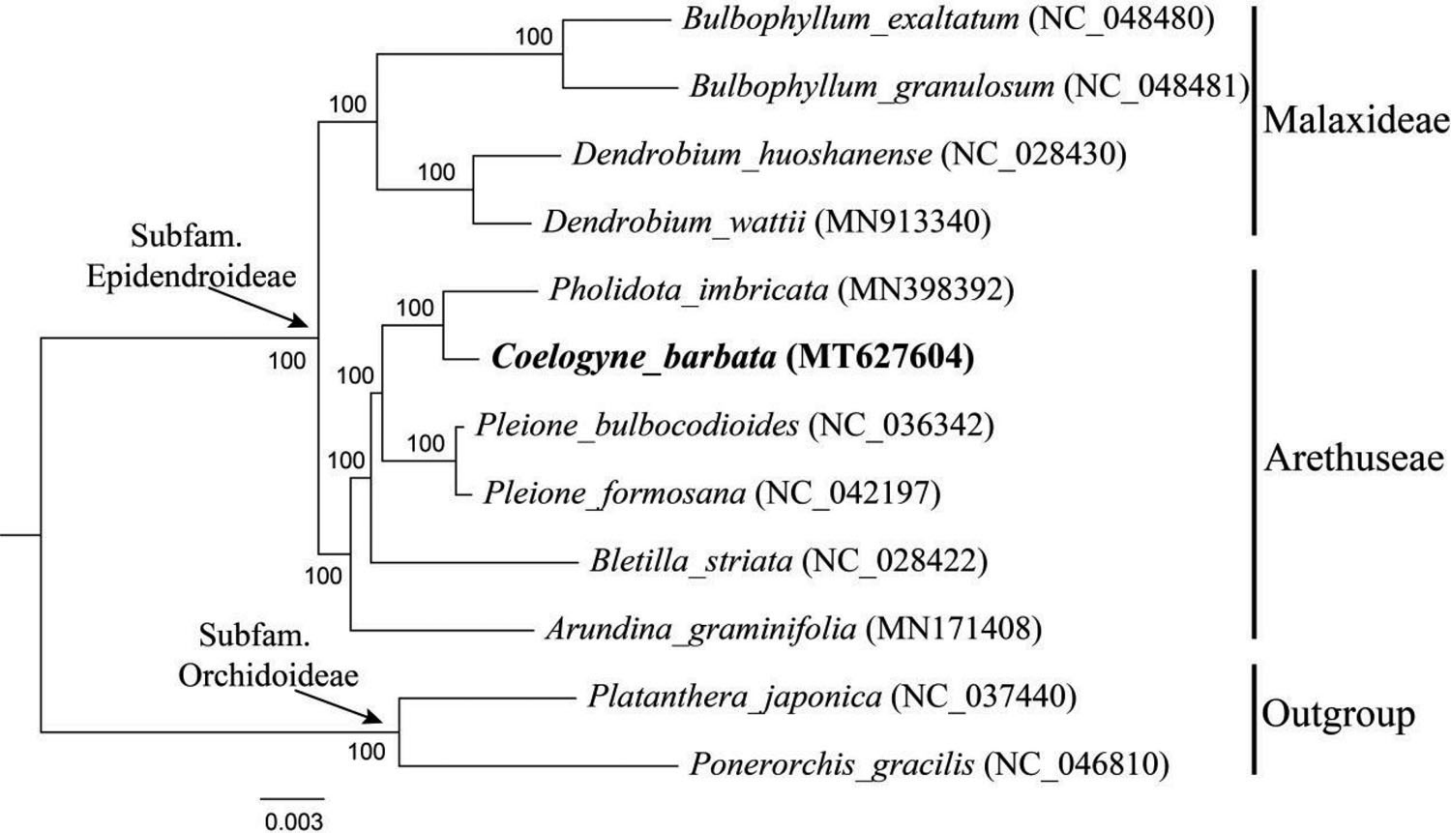
Phylogenetic position of *Coelogyne barbata* inferred by maximum likelihood (ML) based on 79 protein-coding genes from 12 species of Orchidoideae. Sequences used in this study were downloaded from the NCBI GenBank database. The bootstrap values are shown next to the nodes.

## Data Availability

The data that support the findings of this study are openly available in NCBI at https://www.ncbi.nlm.nih.gov, reference number MT627604.
